# A reference-grade chromosome-level genome assembly of a historically important U.S. bread wheat cultivar Timstein

**DOI:** 10.1038/s41597-026-07930-9

**Published:** 2026-07-18

**Authors:** Jatinder Singh, Santosh Gudi, Peter J. Maughan, Sudeshi Seneviratne, Katherine L. D. Running, Gurminder Singh, Upinder Gill, Justin D. Faris, Rajeev Gupta

**Affiliations:** 1 Department of Plant Pathology, Microbiology, and Biotechnology, North Dakota State University, Fargo, ND USA; 2 Cereal Crops Improvement Research Unit, Edward T. Schafer Agricultural Research Center, USDA-ARS Fargo, ND USA; 3 Brigham Young University, Provo, UT USA; 4 Department of Plant Sciences, North Dakota State University, Fargo, ND USA

## Abstract

We report a near telomere-to-telomere high quality genome assembly of the historically important spring wheat cultivar, Timstein, generated using PacBio HiFi long-read sequencing data followed by Hi-C scaffolding. The assembly spanned 14.76 Gb, accounting for all 21 chromosomes of the A, B, and D subgenomes. Gene annotations identified around 105 K high-confidence (HC) gene models. The genome was comprised of ~85% transposable elements, primarily from the Gypsy, Copia, and CACTA families. For each subgenome, the BUSCO completeness score ranging from 97.4 to 99.6% and LTR Assembly Index (LAI) values surpassing 13 indicated the assembly quality was reference grade. Synteny analysis with IWGSC Chinese Spring (CS) RefSeq v2.1 revealed strong chromosomal collinearity between two genomes. Timstein has been extensively studied in classical genetics research for stem and leaf rust resistance and septoria nodorum blotch (SNB) susceptibility. This high-quality genome assembly provides cultivar-resolved references that can expand the wheat pangenome, supports structural and functional genomics studies, and enables fine mapping of disease resistance/susceptibility loci for their utilization in wheat genetics and breeding programs.

## Background & Summary

Wheat (*Triticum aestivum* L.) provides ~20% of the calories for human consumption, and it underpins food security on every continent^1^. As an allohexaploid (2*n* = 6*x* = 42; AABBDD) with a ~16 Gb genome containing > 80% repetitive sequences, bread wheat has posed a challenge for obtaining contiguous, accurate, and complete reference genome assemblies^2,3^. The first high-quality reference for the wheat cultivar Chinese Spring (CS) in 2018 catalyzed a rapid expansion of genomic resources^4^, followed by multiple reference-grade assemblies spanning diverse cultivars and germplasm lines^2,5–11^. Comparative analyses across these genomes have revealed extensive structural variations, introgressions from wild relatives, and presence/absence variations that reflect the complex breeding history of wheat^2,10,12^. Therefore, continued addition of cultivar-resolved references is essential to capture the full spectrum of genetic and functional diversity and to accelerate trait discovery and deployment in wheat improvement programs^12,13^.

Whole genome assemblies provide indispensable frameworks to understand genotype-phenotype relationships, particularly in polyploid species where homeolog redundancy and transposable elements complicate genetic dissection^14^. Chromosome-scale assemblies allow precise localization of quantitative trait loci (QTLs), identification of structural rearrangements, and resolution of gene families that underlie agronomically important traits^15^. However, trait-targeted assemblies, particularly those capturing specific disease resistance, quality, or adaptation phenotypes, remain underrepresented, as most reference genomes are of broadly adapted elite cultivars^10^. Expanding such resources enhances not only the biological relevance of wheat genomics, but also the translational potential of sequence data in breeding programs^15^.

Here, we report a high-quality, chromosome-level genome assembly for the historically important spring wheat cultivar, Timstein, which was released in 1939 and used extensively for wheat breeding, classical genetic studies, and plant pathology studies^16–18^. From the 1950s to 1970s, Timstein was estimated to account for up to 10% of the germplasm in the spring wheat growing areas of North America, and it contributed substantially to breeding programs in CIMMYT and Australia as well^19^. Therefore, Timstein and its derivatives were used for varietal development, particularly in North America, for over three decades. Timstein carries *Sr2*, *Sr11*, *Sr9h, Sr12*, and *Sr16* loci for stem rust resistance and *Lr10*, *Lr23*, *Lr27*, and *Lr31* loci for leaf rust resistance^20–22^. Notably, a pollen killing gene (*ki*) in Timstein is reported to distort the segregation of *Sr11*, making it difficult to study the genetic segregation of this gene^23^. Timstein also harbors the dominant septoria nodorum blotch (SNB) susceptibility gene *Snn7* on chromosome 2D, a key component of the wheat-*Parastagonospora nodorum* pathosystem in which the necrotrophic effector SnTox267 targets multiple *Snn* loci to promote disease^24–26^. A reference-quality Timstein genome will enable the detailed exploration of the *Sr11*-*ki* linkage relationship and dissection of *Snn7*-*SnTox267* molecular interactions. Beyond disease related loci, Timstein also possesses unique grain and end-use quality traits, including kernel hardness and baking absorption^27^, offering additional value to dissect these traits at the genomic level.

**Fig. 1:**
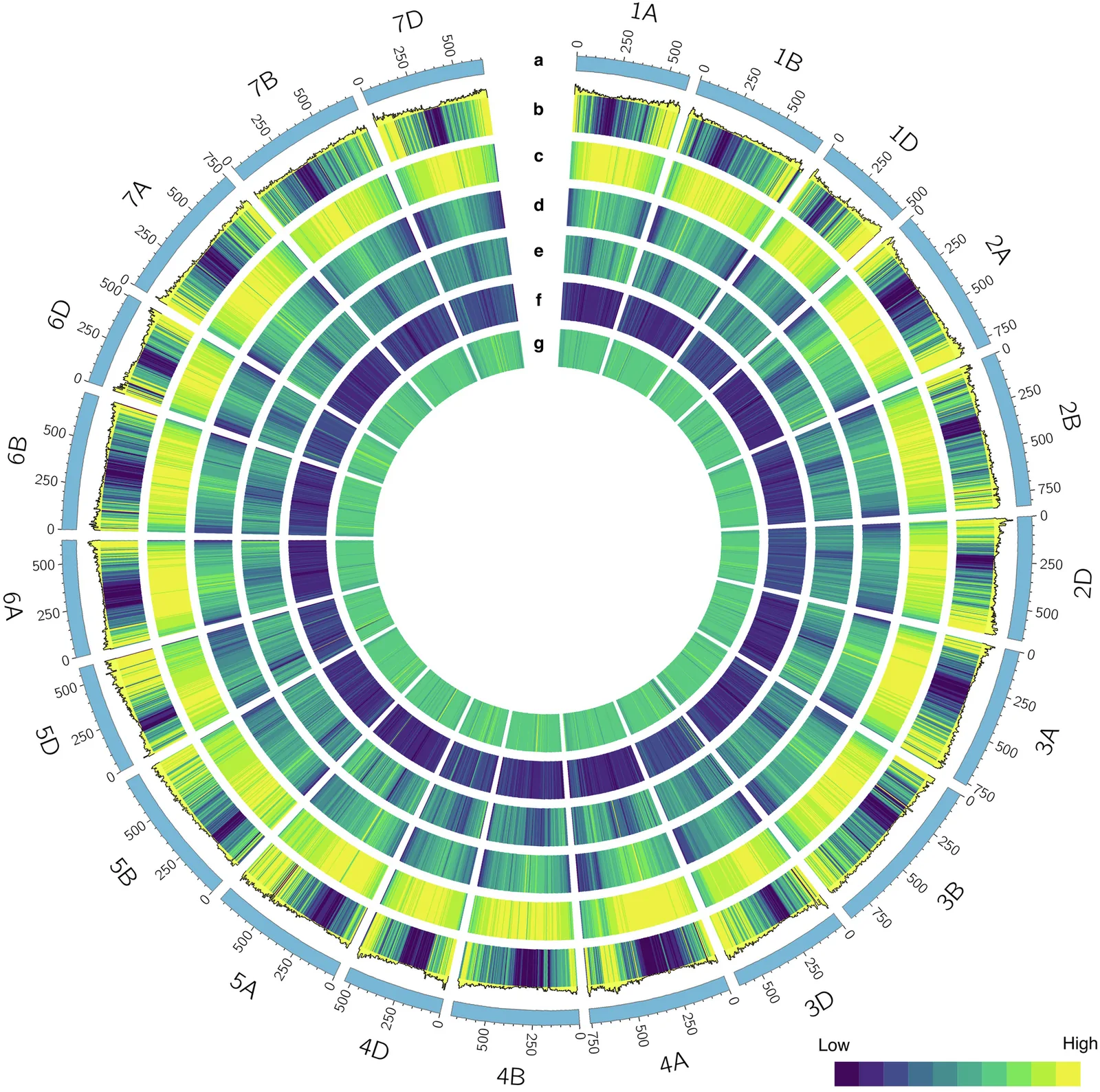
The *circos* plot shows genomic features of Timstein genome assembly. The circles within the plot: **(a)** the individual chromosomes of Timstein assembly with their physical positions (Mb); **(b)** the density (heatmap) and number (histogram) of high-confidence (HC) genes; **(c)** the distribution of the repeat elements over chromosomes; **(d)** the distribution of Gypsy elements; **(e)** the distribution of Copia elements; **(f)** the distribution of CACTA elements; and **(g)** the percentage distribution of GC content (Note: the density plots show the distribution in 5 Mb window.).

**Fig. 2:**
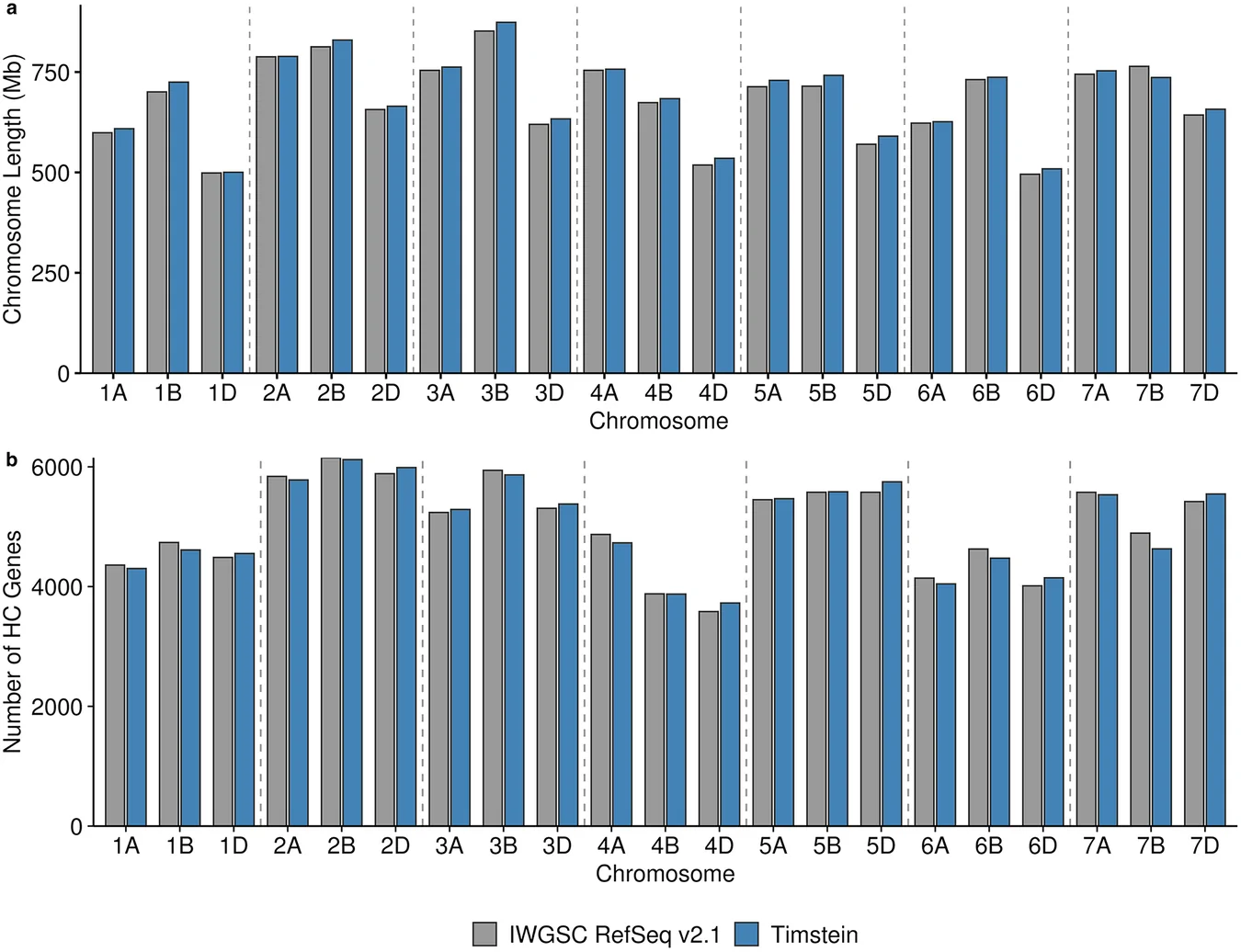
The comparison of **(a)** chromosome length (in Mb) and **(b)** number of high-confidence genes (HC) on three subgenomes (A, B, and D) of IWGSC RefSeq v2.1 and Timstein.

**Fig. 3:**
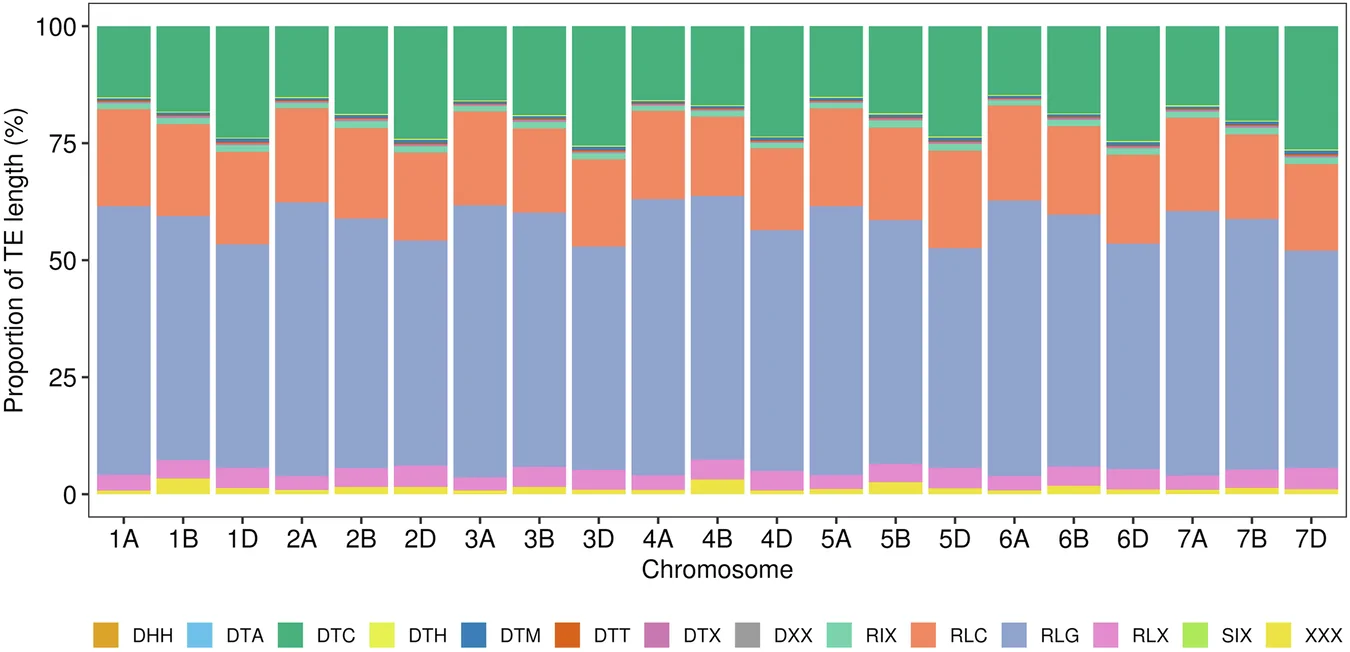
The percentage proportion of different transposable element (TE) superfamilies on 21 chromosomes of Timstein genome assembly.

**Fig. 4:**
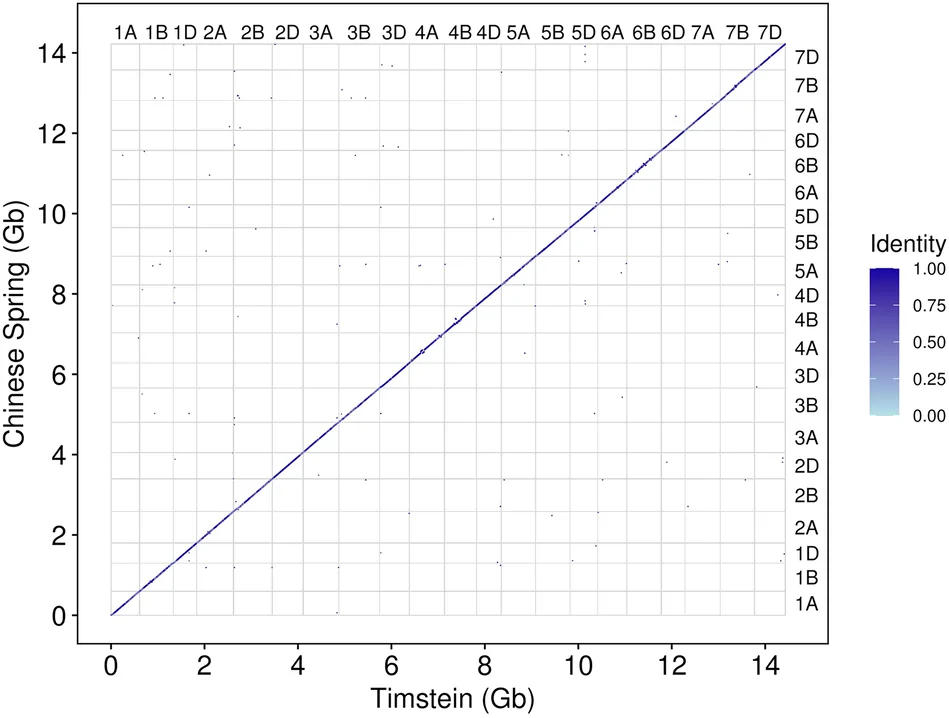
Genome-wide synteny dot plot between Timstein (x-axis) and Chinese Spring (y-axis). Each point represents a sequence alignment, colored by percent identity (light to dark blue indicating increasing similarity). Chromosomes are ordered by subgenome (A, B, and D) and labeled along both axes. The strong diagonal line reflects high collinearity and conserved chromosome structure between the two genomes. Coordinates are shown in Gigabases (Gb).

Using HiFi long-reads and Hi-C data, we assembled the 21 chromosomes of Timstein with an overall genome size of 14.76 Gb (Table 1, Figs. 1a and 2a). There are 105,381 high-confidence (HC) gene models, which are concentrated towards the ends of the chromosomes (Table 2, Figs. 1b and 2b). The telomeric repeat analysis identified canonical telomeric motifs at both ends of 11 chromosomes, while 10 chromosomes contained telomeric repeats at one terminus, indicating near-complete chromosome assemblies. Comparison of different gene annotation metrics with the IWGSC CS RefSeq v2.1 reference genome showed strong concordance, demonstrating the robustness of the gene lift-over approach (Table 2). Analysis of repetitive DNA sequences indicated that ~85% of the genome is composed of transposable elements, dominated by Gypsy (~46%), Copia (~16%), and CACTA (~16%) elements (Table 3, Figs. 1c–f and 3). The GC content was ~ 46% for Timstein genome assembly and is uniformly distributed across the chromosomes (Fig. 1g). Synteny analysis of the Timstein genome assembly with the IWGSC CS RefSeq v2.1 reference assembly showed high conservation across all chromosomes (Fig. 4). This Timstein genome assembly will not only strengthen the foundation of evolutionary and comparative genomics within *T. aestivum* but will also enable high-resolution dissection of complex loci underlying disease resistance/susceptibility and other key agronomic traits in wheat.

**Table 1:** The statistics of Timstein genome assembly.

	Timstein
Total genome size (bps)	14,764,549,218
Total contigs	5,395
Contig N50 (bps)	46,660,406
Contig L50	76
Total chromosomal length (bps)	14,441,404,990
Chromosomes	21
Largest (bps)	873,679,213
N50 (bps)	729,189,812
N90 (bps)	535,110,000
L50	10
L90	19
Total gaps	925
GC content (%)	46.04
BUSCO (%) (A, B, and D subgenomes)	99.1, 97.4, and 99.6
HC genes	105,381
LC genes	159,789
Repeat elements (%)	85.71
k-mer completeness (%)	98.71
QV	65.57
LAI (A, B, and D subgenomes)	16.98, 18.42, 13.49

**Table 2:** Statistics of the annotated gene models of Timstein assembly in comparison with IWGSC CS RefSeq v2.1.

	Timstein	IWGSC CS RefSeq v2.1
Total genes	105,381	105,535
Total transcripts	129,211	131,120
Total exons	725,536	737,687
Average gene length (bps)	3,424	3,497
Median gene length (bps)	2,348	2,389
Average CDS length (bps)	1,328	1,337
Median CDS length (bps)	1,131	1,137
Average exon length (bps)	301	303
Median exon length (bps)	153	153
Average intron length (bps)	479	489
Median intron length (bps)	138	139
Exons per gene	6.88	6.99
Monoexonic genes	26,291	26,032
Multiexonic genes	79,090	79,503
Exons per transcript	5.62	5.63
Monoexonic transcripts	26,531	26,324
Multiexonic transcripts	102,680	104,796
Average transcripts per gene	1.23	1.24

**Table 3:** Summary of transposable element composition in the Timstein genome assembly.

	TE Length	TE Proportion (%)
All TEs	12,378,365,017	85.71%
**Class 1**	9,647,397,967	66.80%
*LTR retrotransposons*		
Gypsy (RLG)	6,633,782,995	45.94%
Copia (RLC)	2,384,335,604	16.51%
Unclassified RT-LTRs (RLX)	466,273,934	3.23%
*Non-LTR retrotransposons*		
LINE (RIX)	161,631,555	1.12%
SINE (SIX)	1,373,879	0.01%
**Class 2**	2,552,925,328	17.68%
*DNA transposons*		
CACTA (DTC)	2,374,467,672	16.44%
Mutator (DTM)	69,538,382	0.48%
Harbinger (DTH)	27,438,163	0.19%
Mariner (DTT)	29,379,163	0.20%
Unclassified with TIRs (DTX)	38,118,401	0.26%
Unclassified Class 2 (DXX)	10,438,672	0.07%
hAT (DTA)	1,586,714	0.01%
Helitrons (DHH)	1,958,161	0.01%
**Unclassified repeats (XXX)**	178,041,722	1.23%

## Methods

### Plant material and DNA extraction

Timstein is a hard red spring wheat cultivar released by the University of Minnesota, Saint Paul, MN, USA, in 1939^24^. Timstein was selected for sequencing because of the relevance and importance of the resistance and susceptibility genes that it possesses, including resistance to stem rust^16^ and leaf rust^17^; and susceptibility to SNB^24^.

The seeds were grown in a hydroponics system in a controlled growth chamber set at 12 hours (h) of day light with temperature settings for day and night as 20 °C and 18 °C, respectively. We used 1.7 g/L MaxiBloom® Hydroponics Plant Food (General Hydroponics, Sevastopol, CA, USA) to grow the plants. The leaf samples were collected from 3-week-old seedlings for high molecular weight (HMW) DNA extraction using a CTAB-Qiagen Genomic-tip method^28^. The quality and quantity of extracted DNA was assessed using nanodrop spectrophotometer and Qubit dsDNA HS assay, respectively.

### DNA sequencing and Genome assembly

For PacBio library preparation, the HMW DNA was sheared to 17 kb fragments using Diagenode Megaruptor. SMRTbell adapter libraries were made using SMRTbell Express Template Prep Kit 2.0 following the manufacturer’s instructions. We used Sage BluePippin size selection kit to choose fragments larger than 10 kb. PacBio HiFi sequencing was performed on the PacBio Revio platform using nine 30 h SMRT Cells with the Sequel II Sequencing Kit 2.0, Sequencing Primer v5, and Sequel Binding Kit 2.2. A total of 374.94 Gb of HiFi sequence data was generated, with an average HiFi read length of 14,997 bp and a median read quality score of Q34 (~99.96% accuracy). Approximately 25x PacBio HiFi genome coverage was generated for wheat cultivar, Timstein. For scaffolding the primary contigs, chromosome conformation capture (Hi-C) data was generated at Phase Genomics (Seattle, WA). The leaf tissue from the same 3-week-old plant used for PacBio sequencing was submitted to Phase Genomics for Hi-C library preparation. Three Hi-C libraries were generated using the standard Phase Genomics proximity ligation workflow, in which crosslinked chromatin was digested using a four-enzyme cocktail (DpnII, DdeI, HinfI, and MseI), proximity ligated and converted into Illumina paired-end sequencing libraries (2 × 150 bp). A total of 434,144,586 Hi-C read pairs were generated for chromosome-scale scaffolding.

To assemble PacBio HiFi reads into contigs, we used hifiasm (v0.24)^29–31^ with default parameters for an inbred species (-l0). The primary PacBio HiFi contig assembly consisted of 5,395 contigs spanning 14.81 Gb, with an average contig length of 2.75 Mb and a largest contig size of 397.66 Mb. The assembly achieved a contig N50 of 46.66 Mb with a contig L50 of 76 contigs, indicating high continuity prior to Hi-C scaffolding. The primary contig assembly contained no gaps prior to scaffolding.

Raw Hi-C reads were aligned to the corrected primary contig assembly using the Arima Mapping Pipeline (A160156_v03, Arima Genomics, Carlsbad, California, USA). Alignment rates were 99.87% and 99.72% for read 1 and 2, respectively. Following alignment, filtering, and duplicate removal, 108,155,414 unique valid Hi-C pairs were retained for scaffolding, including 36,906,599 intra-chromosomal contacts and 71,248,815 inter-chromosomal contacts. Among the intra-chromosomal contacts, 21,200,135 pairs spanned distances greater than 10 kb. Contigs were clustered, ordered, and oriented using YaHS v.1.2a.2^32^, a proximity-guided scaffolder, followed by manual inspection and correction using JuiceBox v2.15^33^. Inter-contig gaps within scaffolds were padded with 100 Ns. The IWGSC CS RefSeq v2.1 reference genome was used to name and orient the pseudo-molecules.

We used blobtools v1.1.1^34^ to identify and separate organellar contigs and remove any other genomic contaminants. The analysis identified 275 mitochondrial contigs totaling 11.48 Mb and 1,227 chloroplast-derived contigs totaling 46.49 Mb, which were assembled using oatk v1.0^35^. Additionally, five short contigs (<15 kb) were excluded from the final assembly. No contigs assigned to non-Poales taxa were detected. In total, 1,507 contigs comprising 58.02 Mb were removed from the final nuclear genome assembly. Further, we performed a telomere search on all the chromosomes using tidk (v0.2.31)^36^ with “clade” option set to “Poales” and to search for “AAACCCT” telomeric repeats. The genome assembly was evaluated for accuracy, completeness, and contiguity, using tools such as Merqury^37^, QUAST (v5.2.0)^38^, assembly-stats (v1.0.1), BUSCO (v 6.0.0)^39,40^, and LTR_retriever (v3.0.4)^41^.

### Transposable element and gene model annotations

For transposable element annotations, we used well curated ClariTeRep database^42^ and masked the repeats with RepeatMasker (v 4.1.5)^43^, using “-xsmall”, “-xm”, and “-engine rmblast” parameters^44,45^. The “.out.xm” file from RepeatMasker was post-processed using clariTE.pl script (https://github.com/jdaron/CLARI-TE).

We used gene lift-over technique to get the gene model annotations from IWGSC CS RefSeq v2.1 (https://urgi.versailles.inra.fr/download/iwgsc/IWGSC_RefSeq_Annotations/v2.1/, accessed Sept 26^th^, 2025). The high-confidence (HC) and low-confidence (LC) gene models from IWGSC CS RefSeq v2.1 were combined, and liftoff (v1.6.3)^46^ was used to annotate the Timstein genome assembly. To maximize annotation completeness and compatibility with existing wheat genomic resources, both HC and LC gene models from IWGSC CS RefSeq v2.1 were included in the lift-over annotation process. The parameters such as 50% alignment coverage (-a 0.5), 70% sequence identity (-s 0.7), no partial mappings (exclude_partial), allow multiple copies (-copies), and polishing (-polish) were used in liftoff and renamed following the IWGSC CS RefSeq v2.1 gene naming convention. For instance, Timstein genes were named as “TraesTS1A01G#######”. The annotated Timstein gene models were compared with IWGSC CS RefSeq v2.1 using liftofftools (v0.4.3)^46^. Because the annotation workflow transferred existing IWGSC CS gene models onto the Timstein assembly, similar annotation statistics between the two genomes were expected and should not be interpreted as direct evidence of biological equivalence between the cultivars. These resulting annotations may not fully capture Timstein-specific genes, structurally divergent loci, or highly variable resistance gene families. Therefore, additional evidence-based annotation approaches may further improve the structural gene annotations of Timstein genome in future studies. Further, the pairwise alignments were computed using minimap2 (v2.30-r1287)^47^ with parameters “-f100 -K8g -cx asm5”. The dotplots were generated using “.paf” files with ggplot2^48^ package in R (4.4.0).

## Data Records

The raw PacBio HiFi sequencing data for Timstein have been deposited in the NCBI Sequence Read Archive (SRA)^49^ database with accession numbers – SRR37600978-SRR37600984, SRR37600987, and SRR37600988. The Hi-C sequencing data for Timstein was submitted to SRA with accession numbers – SRR37600977, SRR37600985, and SRR37600986. We submitted the genome assembly to GrainGenes Genome Browser (https://graingenes.org/GG3/genome_browser)^50^. The genome assembly and supporting datasets, including mitochondrial and chloroplast genomes, repeat annotations, and gene models are available via figshare^51^ in fasta, gff3, protein, CDS, and repeat elements gff3 formats for direct downloading. The chromosome-scale genome assembly is also accessible through GenBank under GCA_058774805.1^52^ as a part of BioProject PRJNA1435230.

## Technical Validation

**Fig. 5:**
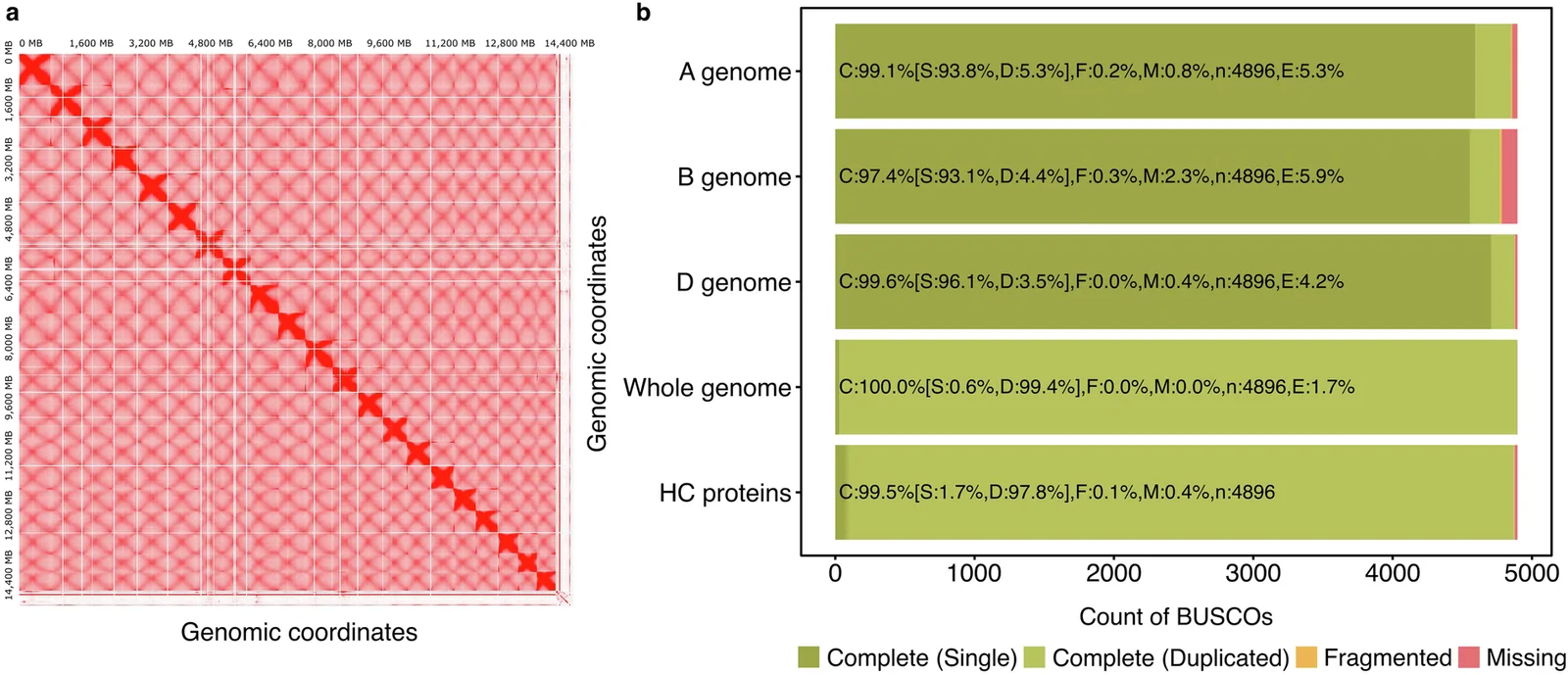
Assembly quality assessment of Timstein genome. **(a)** Hi-C contact map, and **(b)** BUSCO completeness analysis showing gene-space completeness of subgenomes (A, B, and D), whole genome assemblies, and using high-confidence (HC) proteins.

The Hi-C contact maps showed consistent and strong intra-chromosomal interactions, indicating accurate scaffolding and correct chromosome-level assembly (Fig. 5a). The genome assembly had an N50 value of 729.19 Mb, with an L50 value of 10, indicating high continuity (Table 1). The consensus quality value (QV) of 65.57 and k-mer completeness of 98.71% demonstrated the reference-grade quality of the genome assembly. BUSCO analysis using 4896 single-copy orthologs showed 4894 complete genes, indicating near-complete gene space representation, with a mean duplication level of three, as expected for a hexaploid species (Fig. 5b). Moreover, high LTR assembly index (LAI) scores of 16.98, 18.42, 13.49 for A-, B-, and D-subgenomes, respectively, further confirmed the high contiguity and completeness of the Timstein genome assembly (Table 1).

## Usage Notes

To support community use, the genome assembly, gene models, and repeat annotations have been deposited in public repositories. Interactive visualization of genome and annotated gene models are available through GrainGenes genome browser at https://graingenes.org/jb/?data=/ggds/whe-timstein. The BLAST search functionality is available at https://graingenes.org/blast/.

## Data Availability

The genome assembly, raw sequencing data, and annotations have been deposited in public repositories. The data generated in this study are available at NCBI under BioProject accession number PRJNA1435230 (https://www.ncbi.nlm.nih.gov/bioproject/PRJNA1435230/). Raw PacBio HiFi and Hi-C sequencing data for Timstein is accessible through SRA accession numbers from SRR37600977 to SRR37600988 (https://identifiers.org/ncbi/insdc.sra:SRP683282). The genome is available on GrainGenes Browser (https://graingenes.org/jb/?data=/ggds/whe-timstein) and GenBank (https://identifiers.org/ncbi/insdc.gca:GCA_058774805.1). The genome assembly and supporting datasets, including mitochondrial and chloroplast genome, repeat annotations and gene models are also available via figshare (https://doi.org/10.6084/m9.figshare.31633780.v4).

## Code availability

We used publicly available bioinformatics tools for all the analyses in this study. We followed the official documentation and recommended workflows of each software package. Detailed information on software names, versions, and parameters is provided in the Methods section. We used default settings, unless otherwise noted. All command-line procedures and analyses can be reproduced using the listed software and parameters. No custom scripts were developed in this study.
